# Compound Kushen Injection as an Adjunctive Therapy for the Treatment of Non-Small-Cell Lung Cancer: A Meta-Analysis of Randomized Controlled Trials

**DOI:** 10.1155/2019/7241927

**Published:** 2019-11-06

**Authors:** Liu Pu, Wei-hao Chen, Lu-xi Cao, Kun-ji Wu, Shu-lian Chen, Ji-huan Lin, Cheng-lu Li, Shi-qi Wang, Ming-min Zhu, Yi-min Zhang

**Affiliations:** ^1^Traditional Chinese Medical College, Jinan University, 601 Huangpu West Avenue, Guangzhou, Guangdong 510632, China; ^2^Hainan Provincial Hospital of Traditional Chinese Medicine, 47 Heping North Road, Haikou, Hainan 570203, China; ^3^School of Stomatology, Jinan University, 601 Huangpu West Avenue, Guangzhou, Guangdong 510632, China; ^4^Haikou People's Hospital, 43 People's Avenue, Haikou, Hainan 570208, China

## Abstract

**Objectives:**

To evaluate the efficacy and safety of compound Kushen injection (CKI) combined with chemo treatment (chemo) for non-small-cell lung cancer (NSCLC).

**Methods:**

We systematically searched the literature published in seven databases, including Embase, PubMed, central, MEDLINE, CNKI, Wanfang, and VIP, from their inception to April 2019 for all randomized controlled trials (RCTs) comparing CKI plus chemo with chemo alone in patients with NSCLC. Our main end point was clinical efficiency and the secondary outcomes were Karnofsky performance score (KPS), immune function, and adverse events. The Cochrane risk of bias tool was applied for quality assessment.

**Results:**

10 studies involving 1019 participants were included. The clinical response rate (relative risk (RR) = 1.21, 95% confidence interval (CI): 1.06 to 1.37; *P*=0.003), KPS (RR = 2.18, 95% CI: 1.49 to 3.17; *P* < 0.0001), immune function (mean differences (MD) = 0.82, 95% CI: 0.12 to 1.52; *P*=0.02) and adverse effects (RR = 0.67, 95% CI: 0.60 to 0.74; *P* < 0.00001) in the CKI plus chemo group showed significant differences when compared with chemo alone.

**Conclusions:**

CKI combined with chemo can improve clinical efficiency, KPS, and immune function and reduce adverse reactions in patients with NSCLC when compared with chemo alone. However, more rigorously designed RCTs are needed to validate this benefit, as some of the included RCTs are of low methodological quality.

## 1. Introduction

Lung cancer is the leading cause of cancer-related deaths worldwide, of which 80% are NSCLC [1, 2]. In 2012, more than 1.6 million people died of lung cancer, and this number is expected to increase to 3 million by 2035, continuing to be a major health problem [3, 4]. NSCLC is one of the lung cancers with a high clinical incidence [5]. Because most patients with NSCLC have missed the best time for surgery when they are diagnosed, they have to choose chemo [6, 7]. However, the safety and efficacy of chemo are limited, with a series of side effects, such as myelosuppression, nephrotoxicity and neurotoxicity, as well as immunosuppression and acute tissue response [8, 9]. Therefore, developing a drug that can effectively control or alleviate adverse reactions of chemo has always been the goal of clinical treatment.

As a traditional Chinese medicine, CKI has been extensively used in the adjuvant treatment of various kinds of cancers [10], which include NSCLC [11], primary hepatic carcinoma [12], gastric carcinoma [13], and nasopharyngeal carcinoma [14]. Modern pharmacological studies have shown that CKI has multiple effects, including anticancer, anti-inflammatory, analgesic and immune regulation [15]. CKI combined with chemotherapeutic drugs plays an antitumor role by activating the immune system and increasing the activity and quantity of T lymphocyte, thus effectively controlling the growth of tumor cells [16]. However, the efficacy of CKI combined with chemo on NSCLC still lacks systematic evaluation criteria. Therefore, this meta-analysis aimed to investigate whether CKI combined with chemo can improve clinical efficiency, KPS, and immune function and reduce adverse reactions in patients with NSCLC when compared with chemo alone.

## 2. Method

### 2.1. Search Strategy and Selection Criteria

We searched seven databases, including PubMed, Embase, central, MEDLINE, CNKI, Wanfang, and VIP, from the earliest possible year to April 2019, with no language restrictions. Besides, to achieve the maximum sensitivity of the search strategy, we also manually searched the literature published in Chinese or English, using the list of references in the main literature.

### 2.2. Inclusion and Exclusion Criteria

#### 2.2.1. Research Object

All patients enrolled in this study, regardless of gender and age, were diagnosed by histopathological examination and were expected to survive for more than 3 months, as well as in line with the relevant standards for NSCLC developed by the International Union Against Cancer (UICC) in 1997 [17]. Chemotherapy contraindications and other systemic acute diseases affecting the test results were excluded.

#### 2.2.2. Type of Study

In all the included RCTs, the control group was treated with chemo and the experimental group was given CKI on the basis of the control group. In brief, the requirement of the experimental design should reflect the individual effects of CKI. And there were no limits to the treatment dose and duration in both groups. All observational and cohort studies were excluded.

### 2.3. Type of Outcome Measures

The primary outcome was clinical efficiency, and the secondary outcomes were KPS, immune function (CD3^+^, CD4^+^, CD8^+^, CD4^+^/CD8^+^, immunoglobulin A (IgA), immunoglobulin G (IgG), and immunoglobulin M (IgM)), and adverse events including gastrointestinal reaction, the reduction of white blood cell (WBC), neutrophilic granulocyte, blood platelet, and hemoglobin. Depending on the classification and efficacy evaluation criteria of tumor lesions determined by the World Health Organization (WHO), CR (complete remission) and PR (partial remission) were considered clinically effective.

### 2.4. Data Extraction

Data were independently extracted by two reviewers, Liu Pu and Weihao Chen. We preliminarily excluded studies that did not meet the inclusion criteria by reading the titles and abstracts. For documents that could not be adequately judged, we assessed them by reading the full text. In order to avoid subjectivity of the reviewer, the author's name and organization were hidden in the process of evaluation. We resolved disagreements between the two investigators through discussions with the senior researcher Yimin Zhang. The following information were extracted: first author, year of publication, sample, intervention, the dose of CKI, course of treatment, clinical efficacy, KPS, adverse events, and parameters of immune function such as CD3^+^, CD4^+^, CD8^+^, CD4^+^/CD8^+^, IgA, IgG, and IgM.

### 2.5. Study Quality Evaluation

Two evaluators, Kun-ji Wu and Liu Pu, assessed risks including research bias based on the Cochrane bias risk tool. There were six aspects: (1) selection bias (random sequence generation and allocation concealment), (2) performance bias (participant and personnel constraints), (3) detection bias (blind method for evaluation of results), (4) consumption deviation (incomplete date of results), (5) report bias (selective report), and (6) other biases (other potential bias). We have resolved all the differences by achieving a consensus with the third author (Yimin Zhang).

### 2.6. Data Analysis

All statistical data were aggregated and analyzed with Review Manager 5.3 (RevMan 5.3). We used 95% CI to calculate MD and RR for comparing successive and dichotomous variables, respectively. Calculating ways included Cochran's *Q* statistics and *I*^*2*^ statistics. If there was significant heterogeneity (*I*^*2*^ ≥ 50% and *P* < 0.05), the random effect model was used to synthesize the data. Otherwise, the fixed effect model was utilized. If more than 10 studies were included, we would use the funnel plot and Egger's or Harbord's modified test to evaluate the publication.

## 3. Result

### 3.1. Study Selection

**Figure 1:**
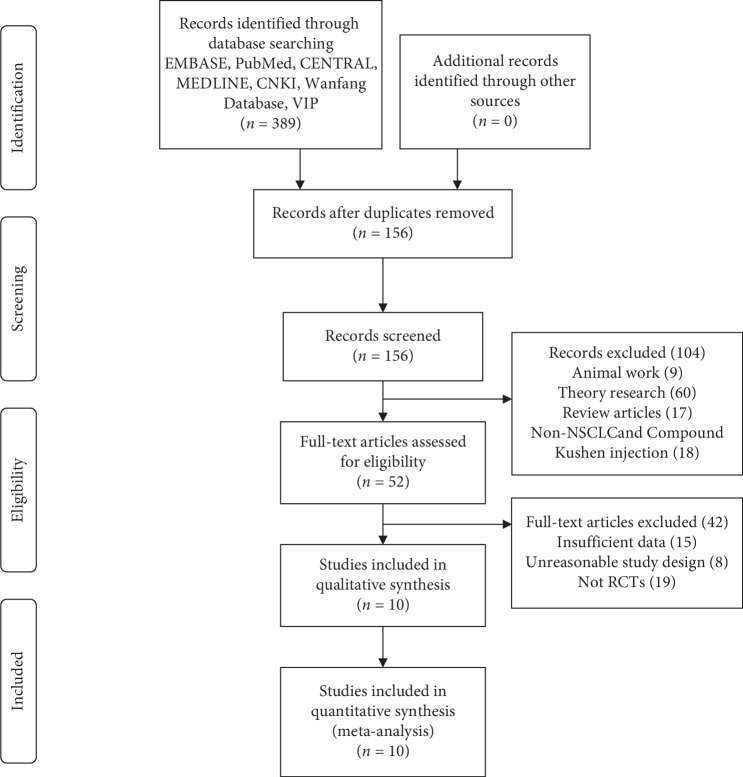
Flow diagram of study selection.

Through the retrieval of electronic databases such as PubMed, Embase, central, MEDLINE, CNKI, Wanfang, and VIP, 389 references were obtained. And after eliminating duplicate documents in EndNote software, 156 articles were retained for further evaluation. First, 104 articles, including 9 animal studies, 60 theoretical studies, 17 reviews, and 18 non-NSCLC and CKI, were removed by reading the titles and abstracts. Second, 42 references were further excluded by reading the full text, of which 15 had incomplete information, 8 were unreasonable in design, and 19 were non-RCTs. Finally, 10 eligible trials [18–27] were identified for appraisal and data extraction (Figure 1).

### 3.2. Study Characteristics

A total of 1019 patients were enrolled in these 10 studies, 505 patients underwent chemo in the control group and 514 patients received CKI combined with chemo in the experimental group. CKI-based therapies were mainly used both in traditional Chinese medicine (TCM) and in integrated Chinese and Western medicine, so all research studies were from China and published in Chinese. And all these studies reported clinical efficacy. Five studies [19, 22–25] described KPS. Seven articles [18–21, 23, 25, 27] reported immunologic function and seven articles [18–20, 22–24, 26] discussed adverse effects. The basic information and details of 10 studies were listed in Table 1.

### 3.3. Primary Outcome Measures

#### 3.3.1. Clinical Efficiency

**Figure 2:**
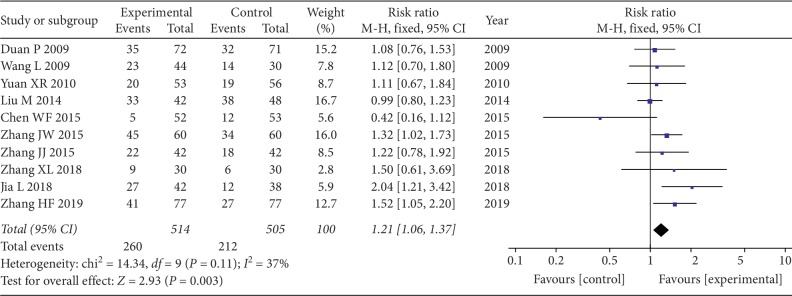
Forest plot of improved clinical response rate.

Ten studies, including 1019 participants, reported clinical effectiveness. The test results of heterogeneity between two studies were not statistically significant (chi^2^ = 14.34, *I*^*2*^ = 37%; *P*=0.11), and the fixed effect model was chosen. The analysis showed that CKI combined with chemo significantly improved clinical efficiency compared with chemo alone in patients with NSCLC (RR = 1.21, 95% CI: 1.06 to 1.37; *P*=0.003) (Figure 2).

### 3.4. Secondary Outcome Measures

#### 3.4.1. KPS

**Figure 3:**
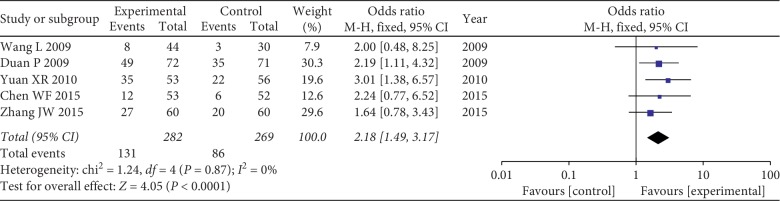
Forest plot of KPS.

Five studies including 551 patients evaluated KPS. The results showed that there was no significant difference between the two groups, and the fixed effect model was selected (chi^2^ = 1.24, *I*^*2*^ = 0%; *P*=0.87). Results showed that CKI combined with chemo significantly improved KPS in patients with NSCLC when compared with chemo alone (RR = 2.18, 95% CI: 1.49 to 3.17; *P* < 0.0001) (Figure 3).

#### 3.4.2. Immune Function

**Figure 4:**
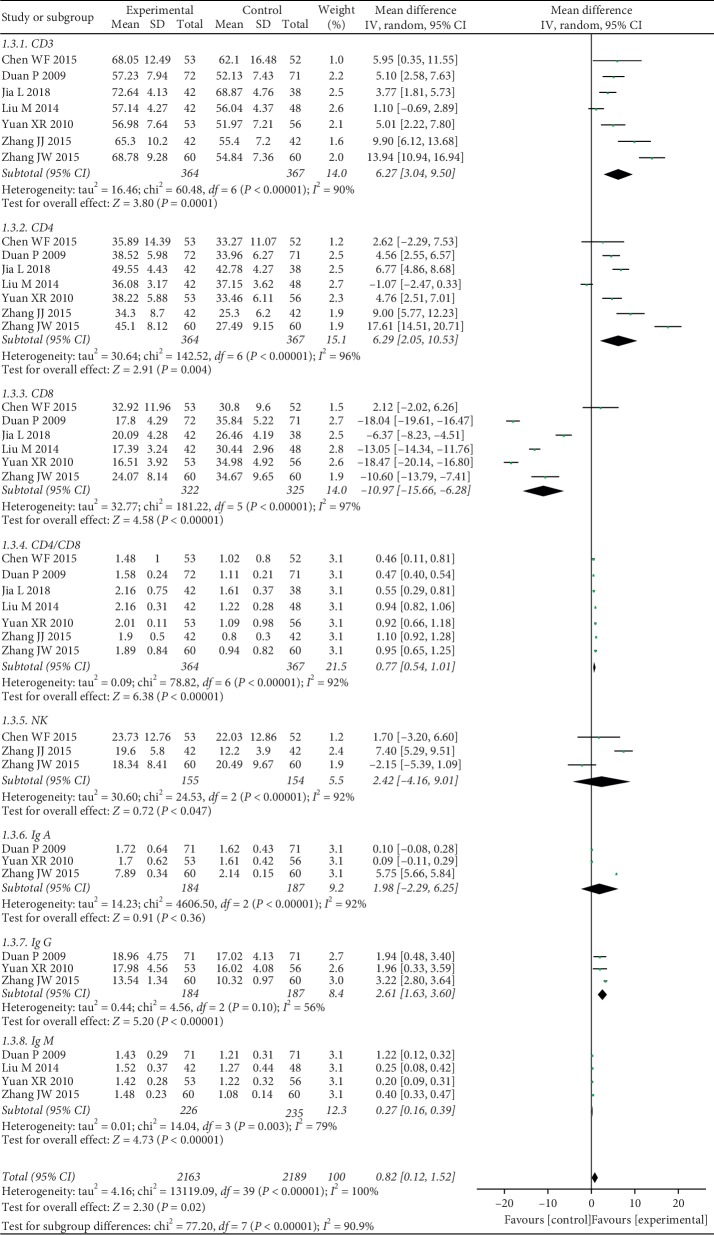
Forest plot of immune function.

Seven studies including 731 patients reported CD3^+^. There was a high degree of difference in heterogeneity between the two groups (chi^2^ = 60.48, *I*^*2*^ = 90%; *P* < 0.00001), and the random effect model was selected for data analysis. The results showed that the combination of CKI and chemo could increase the expression of CD3^+^ more effectively than chemo alone (MD = 6.27, 95% CI: 3.04 to 9.50; *P*=0.0001) (Figure 4).

A total of seven studies involving 731 patients discussed CD4^+^. The heterogeneity test showed a significant difference between the two group (chi^2^ = 142.52, *I*^*2*^ = 96%; *P* < 0.00001). We chose a random effect model to analyze MD and 95% CI. The results indicated that compared with chemo alone, CKI plus chemo could significantly promote the expression of CD4^+^ (MD = 6.29, 95% CI: 2.05 to 10.53; *P*=0.004) (Figure 4).

The expression levels of CD8^+^ were reported in six studies, including 647 cases. The consequences of heterogeneity showed that there was an obvious difference between the two groups (chi^2^ = 181.22, *I*^*2*^ = 97%; *P* < 0.00001). Therefore, we used the random effect model to calculate MD and 95% CI. The results illustrated that there was an obvious difference between two groups, and CKI combined with chemo significantly decreased CD8^+^ when compared with chemo alone (MD = −10.97, 95% CI: −15.66 to −6.28; *P* < 0.00001) (Figure 4).

CD4^+^/CD8^+^ was described in seven studies, including 731 participants. The analysis showed that there was a high heterogeneity between the two groups (chi^2^ = 78.82, *I*^*2*^ = 92%; *P* < 0.00001). The random effect model was applied to synthesize MD and 95% CI, and the results showed that the expression of CD4^+^/CD8^+^ in the group of CKI combined with chemo was significantly up-regulated when compared with chemo alone (MD = 0.77, 95% CI: 0.54 to 1.01; *P* < 0.00001) (Figure 4).

Natural killer cell (NK), one of the parameters in immune function, was described in three studies involving 309 patients. The test results for heterogeneity were of significant differences between two studies (chi^2^ = 24.53, *I*^*2*^ = 92%; *P* < 0.00001). The random effect model was used to estimate MD and 95% CI, and the results showed that there was no significant difference between the two groups (MD = 2.42, 95% CI: −4.16 to 9.01; *P*=0.47) (Figure 4).

IgA was presented in three articles, including 371 patients. There was obvious heterogeneity between the two groups, and the random effect model was used (chi^2^ = 4606.50, *I*^*2*^ = 100%; *P* < 0.00001). There was no significant difference in the results between the two groups (MD = 1.98, 95% CI: −2.29 to 6.25; *P*=0.36) (Figure 4).

There were three studies involving 371 cases discussed IgG. The heterogeneity test showed a moderate difference between CKI and CKI combined with chemo (chi^2^ = 4.56, *I*^*2*^ = 56%; *P*=0.10). We chose a random effect model to analyze MD and 95% CI. The results showed that compared with chemo alone, CKI plus chemo can significantly promote the expression level of IgG (MD = 2.61, 95% CI: 1.63 to 3.60; *P* < 0.00001) (Figure 4).

Four studies with 461 patients reported IgM. The results of heterogeneity were noticeable between two groups (chi^2^ = 14.04, I^2^ = 79%; *P*=0.003). The random effect model was applied for analysis. The results showed that CKI plus chemo was more effective in increasing the expression of IgM than chemo alone (MD = 0.27, 95% CI: 0.16 to 0.39; *P* < 0.00001) (Figure 4).

All these references, including 731 cases, reported immune function. There was considerable heterogeneity between the two groups (chi^2^ = 13119.09, *I*^*2*^ = 100%; *P* < 0.00001). The random effect models were utilized for these studies. The results showed that when compared with chemo alone, CKI combined with chemo dramatically improved immune function in patients with NSCLC (MD = 0.82, 95% CI: 0.12 to 1.52; *P*=0.02) (Figure 4).

#### 3.4.3. Adverse Event

**Figure 5:**
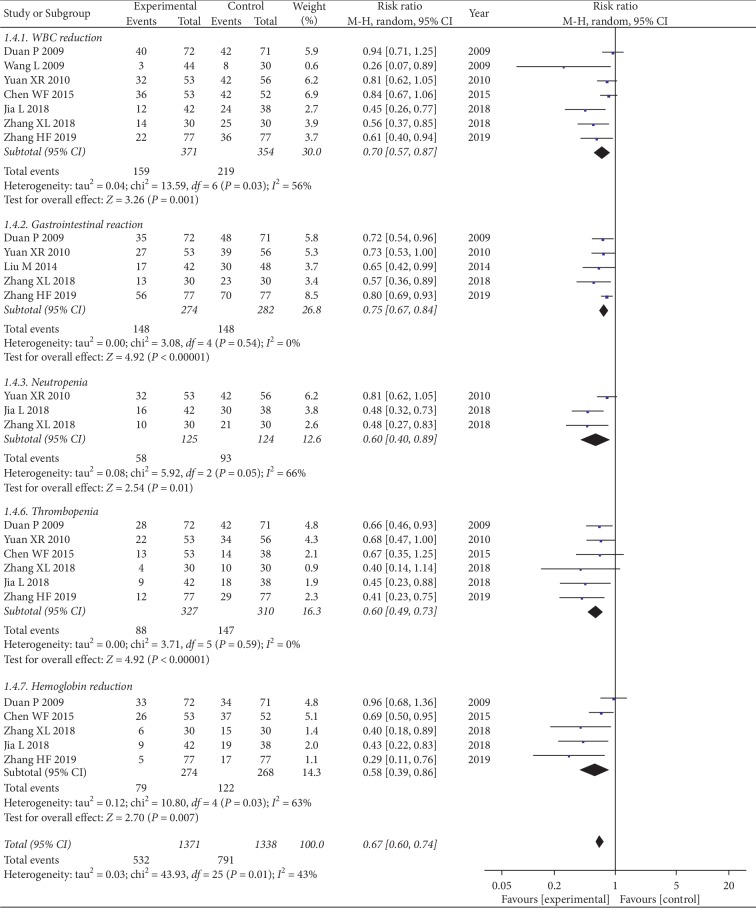
Forest plot of adverse effects.

Seven studies with 725 patients reported WBC reduction. The results represented some evidence of heterogeneity between the two groups (chi^2^ = 13.59, *I*^*2*^ = 56%; *P*=0.03). The results revealed that CKI combined with chemo significantly alleviated the symptoms of leukopenia on patients with NSCLC when compared with chemo alone (RR = 0.70, 95% CI: 0.57 to 0.87; *P*=0.001) (Figure 5).

Three studies involving 249 participants reported neutropenia. Some heterogeneity was found between the two groups (chi^2^ = 5.92, *I*^*2*^ = 66%; *P*=0.05). The results showed that compared with chemo alone, CKI plus chemo markedly decreased the occurrence of neutropenia in patients suffering from NSCLC (RR = 0.60, 95% CI: 0.40 to 0.89; *P*=0.01) (Figure 5).

Five studies with 556 cases reported gastrointestinal adverse reactions. No heterogeneity was observed (chi^2^ = 3.08, *I*^*2*^ = 0%; *P*=0.54). As the results showed, the number of incidents with gastrointestinal discomfort was significantly reduced in the group of CKI combined with chemo when compared with chemo alone (RR = 0.75, 95% CI: 0.67 to 0.84; *P* < 0.00001) (Figure 5).

Adverse effects of thrombopenia were reported in 6 references including 637 cases. There was no any heterogeneity between the two groups (chi^2^ = 3.71, *I*^*2*^ = 0%; *P*=0.59). As illustrated in the results, there was a discernible statistical difference between the two groups. Compared with chemo alone, CKI combined with chemo effectively improved thrombocytopenia in patients with NSCLC (RR = 0.60, 95% CI: 0.49 to 0.73; *P* < 0.00001) (Figure 5).

Five studies including 542 patients discussed hemoglobin. The heterogeneity was clear and there were considerable statistical differences between the two groups (chi^2^ = 10.80, *I*^*2*^ = 63%; *P*=0.03). It was found that CKI plus chemo drastically reduced the number of patients with low hemoglobin when compared with chemo alone (RR = 0.58, 95% CI: 0.39 to 0.86; *P*=0.007) (Figure 5).

Seven studies compared adverse events between patients who underwent CKI plus chemo and those who underwent chemo alone. Heterogeneity between two studies was not observed (chi^2^ = 43.93, *I*^*2*^ = 43%; *P*=0.01). The results showed that compared with chemo alone, CKI combined with chemo significantly reduced adverse events in patients with NSCLC (RR = 0.67, 95% CI: 0.60 to 0.74; *P* < 0.00001) (Figure 5).

### 3.5. Risk of Bias

**Figure 6:**
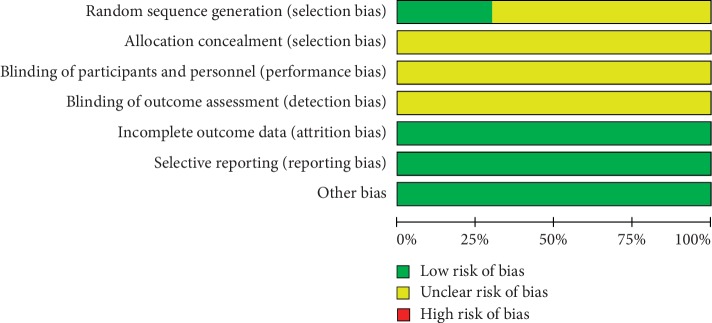
Risk of bias graph.

**Figure 7:**
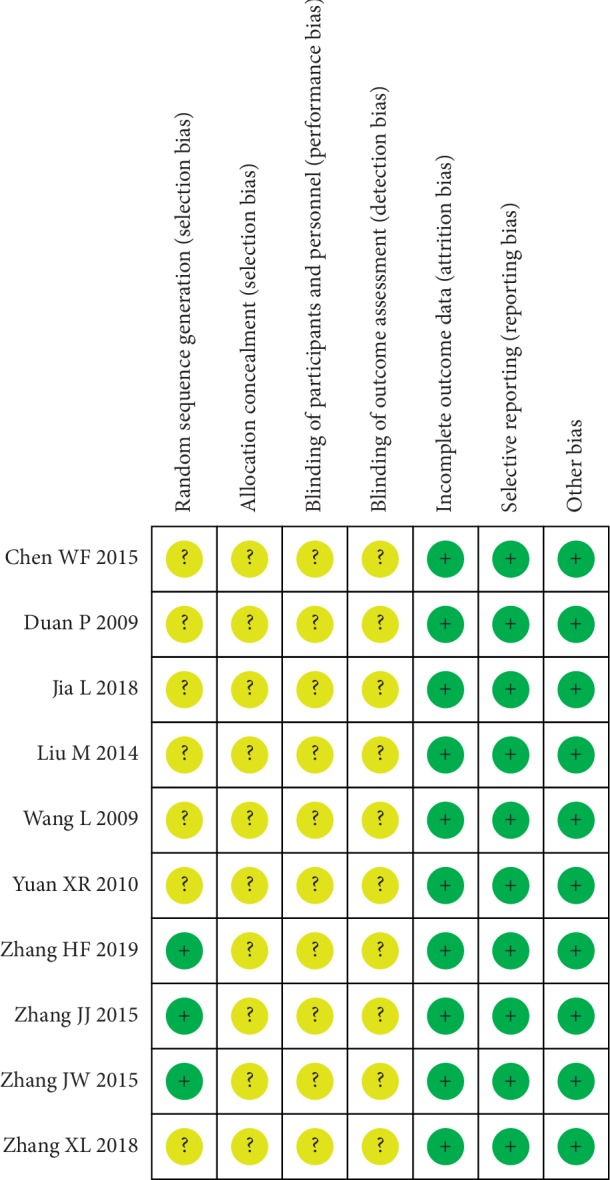
Risk of bias summary of the included studies.

In these studies, all references mentioned randomization, three of which explained specific random methods. Studies that used specific stochastic methods were classified as low risk, while those that only mentioned random but did not use specific random methods were defined as unknown risks. No selection bias, performance bias, and detection bias were reported in the included studies. All studies provided complete data and no selective bias and other biases were discovered (Figures 6 and 7).

### 3.6. Publication Bias

**Figure 8:**
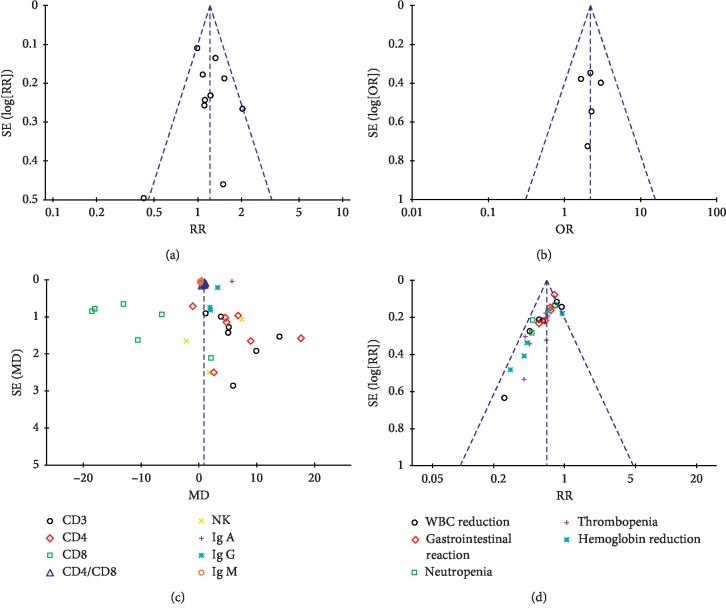
Funnel plot. (a) Funnel plot of clinical efficiency. (b) Funnel plot of KPS. (c) Funnel plot of immune function. (d) Funnel plot of adverse events.

The funnel plot was used to analyze publication bias of the included references. According to Harbord's test, no potential publication bias was found in clinical efficacy (*P*=0.940 > 0.05) and immune function (*P*=0.296 > 0.05), while significant publication bias was found in adverse events (*P*=0.000 < 0.05). Besides, the egger analysis showed no bias in KPS (*P*=0.325 > 0.05) (Figure 8).

## 4. Discussion

The meta-analysis of 10 studies including 1019 patients comparing CKI plus chemo with chemo alone showed that CKI combined with chemo could enhance immune function, upregulate KPS, and reduce adverse events.

Cellular immunity mediated by T lymphocytes is accomplished by delayed hypersensitivity CD4^+^ and cytotoxic CD8^+^, which plays a major role in antitumor. After antigen recognition, activation, and proliferation, CD4^+^ cells synthesize and secrete interleukin-2 (IL-2), human interferon-C (IFN-C), and tumor necrosis factor (TNF), which can dissolve and directly kill tumor cells by recognizing and binding antigens on tumors through antigen receptors. IL-2 promotes the activation and proliferation of T lymphocytes and produces cytokines such as IFN-C and TNF-*β*, which can indirectly kill tumor cells and induce the production of T lymphocytes and NK [28]. Natural killer cells with the function of immune surveillance are lymphocytes that kill tumor cells without the involvement of specific antibodies or expression of MHC-1 or MHC-2 molecules on target cells. Moreover, CD3^+^ is a common marker of all T lymphocytes, and the downregulation of its expression can lead to immune imbalance. In summary, the balance between T cell subsets is a key link to maintain the internal stability of the immune system [29]. However, as the most common treatment for NSCLC, chemo can induce systemic immunosuppression, thus inhibiting the differentiation and maturation of CD3^+^ and CD4^+^ T lymphocytes, resulting in the decrease of CD3^+^ and CD4^+^ T lymphocytes and NK, as well as the imbalance between CD4^+^ and CD8^+^ [30, 31]. Our results showed that CKI combined with chemo can significantly increase CD3^+^ and CD4^+^/CD8^+^ compared with chemo alone, suggesting that CKI can improve the chemotherapy-induced immunosuppression by regulating the expression of T lymphocyte subsets, thereby enhancing the immune function of patients with NSCLC.

The main active ingredients of CKI (Approval No. WS3-B-2752-2004) are matrine, oxymatrine, dehydromatrine, and saponin. In the theory of traditional Chinese medicine, they have the effect of removing pathogenic fever and toxic substances in the blood, as well as clearing heat and diuresis and relieving pain [32]. Studies [33] have demonstrated that exposure to chemotherapeutic drugs can stimulate some signaling pathways in the tumor microenvironment, leading to cancer cell resistance to apoptosis and promoting angiogenesis and tumor growth. Some studies have shown that CKI can effectively block the circulation of Lewis cells in G0/G1 phase, thereby significantly reducing proliferation rate and inducing apoptosis of lung cancer cells [34, 35]. Wang et al. [36] found that CKI can reduce angiogenesis and inhibit tumor growth in tumor tissues. It is suggested that CKI can reduce the side effects of chemo by reducing proliferation [37], promoting tumor cell apoptosis [38], and delaying the rate of tumor angiogenesis [39].

Matrine is the main active ingredient of CKI and has a wide range of pharmacological effects, especially in the field of antitumor. Huang and Xin [40] found that endogenous reactive oxygen species (ROS) contribute to the metastasis of cancer cells; however, matrine can downregulate ROS through ROS/NF-*κ*B/MMPs signaling pathway, thereby inhibiting the migration and invasion of cancer cells. Yi et al. [41] found that matrine can inhibit the proliferation of osteosarcoma cells in vitro and in vivo and inhibit the metastasis of human osteosarcoma cells by downregulating the ERK-NF-*κ*B signaling pathway. Niu et al. [42] reported that matrine can inhibit the proliferation of A549 and 95D cells in lung cancer patients and induce apoptosis by inhibiting Akt in PI3K/Akt/m-TOR signaling pathway and downregulating apoptosis protein inhibitors. In addition, matrine could also induce mitochondrial apoptosis in cisplatin-resistant NSCLC cells by inhibiting the *β*-catenin/survive signaling pathway [43]. What is more, matrine has a significant auxiliary effect on NK and can significantly improve cellular immune function [44, 45]. Therefore, matrine can inhibit tumor cytotoxicity by regulating different signaling pathways, thereby reducing leukopenia, thrombocytopenia, gastrointestinal reaction, and other adverse reactions caused by chemo.

In conclusion, the results of this study indicate that CKI has a certain auxiliary effect on the clinical treatment of NSCLC, which can reduce the side effects caused by chemo and has certain guiding significance for future treatment. However, our research has certain limitations. First, the methodological quality of the research is generally poor. Although most of the included studies involve random methods, only three studies describe specific random methods. There are no references to allocation concealment and blinding, as well as assessment of personnel and outcomes in all included trials. Second, all of the included studies are published in Chinese and may lead to racial bias. It is necessary to include more diverse demographic samples in this meta-analysis to bring richer, more reliable results. Therefore, in view of the limitations of this study, the need for high methodological quality, good experimental design, and large sample size RCTs are needed to study the clinical efficacy and safety of CKI in the treatment of NSCLC.

## 5. Conclusions

In conclusion, compared with chemo alone, CKI combined with chemo can improve the clinical efficacy, KPS, and immune function and reduce adverse reactions in patients with NSCLC. However, given the low quality of the included studies, more rigorous design and large-scale RCTs are needed to validate these conclusions.

## Figures and Tables

**Table 1:** Characteristics of the 10 included studies.

Study (year)	*E*/*C*	Intervention	CKI dosage (ml/day)	Duration	Outcome measures
Duan P. 2009	72/71	CKI + GP vs. GP	20	NA	➀➁➂➃
Wang L. 2009	44/30	CKI + DP/GP vs. TP/GP	20	15–20 days/course, 2 courses	➀➁➂➃
Yuan X. R. 2010	53/56	CKI + GEM vs. GEM	20	4weeks/course, 4 courses	➀➁➂➃
Liu M. 2014	42/48	CKI + NP vs. NP	20	4 weeks/course, 4 courses	➂➃
Zhang J. J. 2015	42/42	CKI + TP vs. TP	20	3 weeks/course, 3 courses	➀➂
Zhang J. W. 2015	60/60	CKI + GP vs. GP	2	3 weeks/course, 2 courses	➀➁➂
Chen W. F. 2015	52/53	CKI + GP/GP/TP vs. GP/GP/TP	20	12 days/course, 2 course	➀➁➂➃
Zhang X. L. 2018	30/30	CKI + TEG vs. TEG	20	3 weeks/course, 4 courses	➀➃
Jia L. 2018	42/38	CKI + PP vs. PP	20	21 days/course, 1 course	➀➁➂➃
Zhang H. F. 2019	77/77	CKI + TP vs. TP	20	21 days/course, 2 courses	➀➁➃

Note. *E*: experimental group; *C*: control group; CKI: compound kushen injection; NA: not available; DP: docetaxel + cisplatin; GP: gemcitabine + cisplatin; GEM: gemcitabine; NP: navelbine + cisplatin; PP: pemetrexed + carboplatin; TP: Taxol + cisplatin; TEG: tegafur; ➀: clinical efficiency; ➁: Karnofsky performance score; ➂: immune function; ➃: adverse reaction.

## Data Availability

The data used to support the findings of this study are included within the article.
